# MOGAD: A Shifting Landscape—From Pathogenesis to Personalised Management, Global Perspectives and Latin American Insights

**DOI:** 10.3390/biomedicines13102344

**Published:** 2025-09-25

**Authors:** Ethel Ciampi

**Affiliations:** 1 Departamento de Neurología, Escuela de Medicina, Pontificia Universidad Católica de Chile, Santiago 8330077, Chile; elciampi@uc.cl or ethelciampi@gmail.com; 2 Red de Salud UC CHRISTUS, Diagonal Paraguay 362, Santiago 8330077, Chile; 3 Neurología, Hospital Dr Sotero del Rio, Santiago 8320000, Chile

**Keywords:** myelin oligodendrocyte glycoprotein antibody-associated disease, MOGAD, pathophysiology, epidemiology, treatment, prognosis, Latin America, LATAM

## Abstract

Myelin oligodendrocyte glycoprotein antibody-associated disease (MOGAD) has emerged as a distinct autoimmune demyelinating disorder, characterised by clinical, radiological, and immunopathological features that differentiate it from Multiple Sclerosis (MS) and AQP4+ neuromyelitis optica spectrum disorder (AQP4+NMOSD). This review provides a comprehensive synthesis of the evolving landscape of MOGAD, from its immunopathogenesis and diagnostic criteria to treatment strategies and global epidemiological insights. We explore the role of MOG-IgG antibodies in disease mechanisms, the utility of emerging biomarkers, and the prognostic value of tools like clinical scores or longitudinal MOG-IgG assessment. Special attention is given to regional disparities, with a focus on Latin America, highlighting diagnostic delays, access inequities, and unique clinical phenotypes. We also examine the limitations of current evidence, including gaps in long-term longitudinal follow-up and variability in diagnostic testing. Finally, we discuss global collaborative efforts and clinical trials that are shaping the future of personalised care in MOGAD. As the field advances, integrating biomarker-driven monitoring, equitable access to therapies, and regionally adapted guidelines will be essential to improving outcomes for patients worldwide.

## 1. Introduction: The Evolving Understanding of MOGAD

Myelin oligodendrocyte glycoprotein antibody-associated disease (MOGAD) has been increasingly recognised as a distinct Central Nervous System (CNS) demyelinating disorder over the past decade, separate from multiple sclerosis (MS) and Aquaporin-4 antibody-positive neuromyelitis optica spectrum disorder (AQP4+NMOSD). Characterised by the presence of immunoglobulin G (IgG) autoantibodies targeting the MOG protein on the surface of oligodendrocytes, MOGAD encompasses a broad clinical spectrum that includes optic neuritis (ON), transverse myelitis (TM), acute disseminated encephalomyelitis (ADEM), and cerebral cortical encephalitis, among others [1,2].

The recognition of MOGAD as a nosologically distinct entity has been driven by advances in cell-based assays (CBAs) for MOG-IgG detection, culminating in the publication of international consensus diagnostic criteria in 2023. These criteria have enabled more accurate diagnosis and have clarified the immunopathological mechanisms underlying the disease, which differ significantly from those in MS and AQP4+NMOSD. Unlike AQP4+NMOSD, MOGAD is not associated with astrocytopathy but rather with antibody-mediated demyelination and complement activation targeting oligodendrocytes [1].

The estimated prevalence of MOGAD is 2.5/100,000 people, with an annual incidence of 3/1,000,000 people [3]. Epidemiologically, MOGAD affects both pediatric and adult populations, with a bimodal age distribution and a less pronounced female predominance compared to other autoimmune demyelinating diseases. The disease course can be monophasic or relapsing, with relapses often involving the same anatomical region as the initial attack. Importantly, MOGAD exhibits a more favourable recovery profile than AQP4+NMOSD, although relapsing forms may lead to cumulative disability [3].

This article aims to synthesise current knowledge on MOGAD, from its immunopathogenesis and clinical heterogeneity to diagnostic strategies and therapeutic approaches. We also highlight regional insights from Latin America, which reveal unique challenges in diagnosis and access to care. Finally, we explore emerging biomarkers, predictive tools, and ongoing clinical trials, as well as global collaborative efforts, that are shaping the future of personalised management in MOGAD.

## 2. Pathophysiology of MOGAD: Beyond Antibodies

MOG is a crucial component of the CNS, functioning as a transmembrane glycoprotein expressed on the surface of oligodendrocytes and extracellular myelin sheaths. Its role extends to maintaining myelin integrity and facilitating adhesion between myelin layers. MOGAD is characterised by the presence of IgG1 autoantibodies targeting MOG. While these autoantibodies are central to the diagnosis and pathology of MOGAD, emerging research indicates a complex interplay of immune mechanisms that extend beyond mere autoantibody presence. More extensive and current reviews concerning the pathogenesis of MOGAD have been published, which extend beyond the scope of the present article [4,5].

### 2.1. Immunopathological Mechanisms

The immunopathogenesis of MOGAD involves both humoral and cellular immune responses. Circulating MOG-IgG antibodies are thought to arise through mechanisms such as molecular mimicry or bystander activation, potentially triggered by infections or environmental factors. These antibodies can cross the blood–brain barrier (BBB), bind to MOG on oligodendrocytes, and initiate a cascade of immune-mediated injury [4,6,7,8].

### 2.2. MOG-IgG Mediated Pathology

The primary pathogenic mechanism in MOGAD involves MOG-IgG-mediated demyelination. MOG-IgG antibodies bind bivalently to MOG on the oligodendrocyte surface, triggering complement activation, antibody-dependent cellular cytotoxicity (ADCC), and enhanced cognate T-cell CNS infiltration and activation. This leads to the lysis of oligodendrocytes and the subsequent loss of myelin sheaths [9,10]. While both MOGAD and AQP4+NMOSD involve complement activation, MOG-IgG may engage C1q less efficiently than AQP4-IgG, potentially leading to less overall complement activation in MOGAD [11]. Histopathological studies of brain biopsies from MOGAD patients show infiltration of T-cells, activated macrophages, and microglia, alongside significant IgG and complement deposition. A distinct feature in MOGAD is the predominance of CD4+ T-cells, contrasting with the CD8+ T-cell presence typically seen in MS [4,5,12].

### 2.3. Cellular Immunity and Cytokine Involvement

Beyond direct antibody effects, cellular immunity plays a significant role. The activation and differentiation of MOG-specific B- and T-cells are crucial for MOG-IgG synthesis. Preceding infections, reported in 20–57% of MOGAD cases, are thought to facilitate antigen recognition and disrupt self-tolerance through mechanisms like molecular mimicry, bystander activation, or exposure of normally sequestered MOG antigens in the CNS. T-cell responses are also implicated. Studies have shown increased Th2 and Th17 cells in MOGAD patients following stimulation with recombinant human MOG protein. Elevated levels of various cytokines and chemokines, including IL-6, GM-CSF, IFN-γ, and IL-10, have been observed in the cerebrospinal fluid (CSF) of MOGAD patients. These inflammatory mediators contribute to the differentiation of CD4+ T-cells into Th17 cells and the production of immunoglobulins. Dysregulation of the immune balance between follicular helper T (Tfh) and follicular regulatory T (Tfr) cells is also hypothesised to contribute to MOGAD pathogenesis, with increased Tfh cells and decreased Tfr cells observed during MOGAD attacks [6,7,8,13].

### 2.4. Oligodendrocyte Cytoskeleton Disruption

An alternative model of MOG-IgG-induced demyelination involves the disruption of the oligodendrocyte cytoskeleton. MOG-IgG binding to MOG can induce crosslinking and repartitioning of MOG within lipid rafts, leading to cellular morphological alterations, including the retraction of myelin-like membrane sheets and cellular processes. This process is associated with a marked disorganisation of F-actin and β-tubulin networks within oligodendrocytes, ultimately reducing their capacity to myelinate axons. This mechanism highlights a direct impact of MOG-IgG on the structural integrity and function of oligodendrocytes, independent of direct cell lysis [4,5,9,10,11,14,15].

### 2.5. Distinctive Features Compared to MS and NMOSD

Histopathological studies have shown perivenous demyelination with relative preservation of axons and astrocytes, distinguishing MOGAD from AQP4+NMOSD, which is characterised by astrocytopathy. Compared to AQP4+NMOSD, MOGAD shows less necrosis and a more favourable response to corticosteroids [16,17]. Unlike MS, MOGAD appears to lack chronic active lesions, cortical demyelination, and oligoclonal bands in most cases. The absence of astrocyte loss and the presence of MOG-IgG as a pathogenic driver underscore its unique immunological identity [4,5,18].

In summary, the pathophysiology of MOGAD extends beyond the mere presence of MOG-IgG autoantibodies. It encompasses a complex interplay of antibody-mediated cytotoxicity, complement activation, dysregulated cellular immune responses, and direct structural disruption of oligodendrocytes, all contributing to the inflammatory demyelination characteristic of the disease.

## 3. Clinical Spectrum and Diagnostic Evolution

MOGAD presents with a broad and age-dependent clinical spectrum, often mimicking other demyelinating disorders such as MS and AQP4+NMOSD. This phenotypic overlap can delay diagnosis and complicate management, making the recognition of MOGAD’s distinct clinical features essential for timely and accurate identification.

### 3.1. Core Clinical Syndromes

The six core clinical syndromes of MOGAD reflect its diverse neurological involvement. Optic neuritis (ON) is the most common presentation in adults, occurring in 50–60% of cases. It is frequently bilateral and characterised by severe visual loss and optic disc oedema. Despite the severity of initial symptoms, visual recovery in MOGAD-ON is generally more favourable than in AQP4+NMOSD. Transverse myelitis (TM) is another hallmark feature, typically presenting as longitudinally extensive lesions with conus medullaris involvement and a predilection for grey matter. Patients often experience motor, sensory, and autonomic dysfunction. Some patients may present with clear symptoms and neurological signs consistent with transverse myelitis, yet show no evident lesion on MRI. This phenomenon can be explained by imaging performed too early—before lesions become radiologically apparent—or by transient lesions that resolve quickly, leaving only a narrow window for detection. In many cases, MRI findings may normalize after the clinical attack. A small proportion of patients may never show any lesion on MRI, which underscores the importance of clinical judgment. In such scenarios, clinicians must carefully differentiate between a true relapse and a pseudorelapse in patients already diagnosed with MOGAD, or consider MOGAD as a potential diagnosis in patients presenting with evident transverse myelitis but negative MRI findings [18,19]. In pediatric populations, acute disseminated encephalomyelitis (ADEM) is the predominant presentation, affecting 40–50% of children. It is marked by multifocal white matter lesions and encephalopathy. Brainstem syndromes, including ataxia, diplopia, and vertigo, are also observed and may overlap with other core features. Cerebral cortical encephalitis, sometimes fulfilling criteria for FLAMES (FLAIR-hyperintense Lesions in Anti-MOG-associated Encephalitis with Seizures), presents with seizures and cortical lesions. Finally, cerebellar syndromes, though less common, are increasingly recognised in both adult and pediatric cohorts [1,2,3,20,21].

### 3.2. Age-Related Phenotypic Variability

The clinical phenotype of MOGAD varies significantly with age. In children, ADEM is the hallmark presentation, often accompanied by encephalopathy and seizures [20,21,22]. In contrast, adult MOGAD is dominated by ON and TM, with additional manifestations such as cortical encephalitis and brainstem involvement [1,2,3]. Seizures occur in approximately 10–15% of adult cases. Late-onset MOGAD, defined as disease onset after age 50, accounts for 15–25% of cases. These patients frequently present with ON and cerebral symptoms and are more likely to have comorbid autoimmune or vascular conditions, which can obscure diagnosis and complicate treatment [3,22,23,24] (Table 1).

### 3.3. Diagnostic Evolution

The publication of the 2023 International Panel Consensus Criteria for MOGAD marked a significant advancement in diagnostic standardisation [1]. These criteria emphasise the presence of a core clinical event, combined with a positive MOG-IgG result obtained via cell-based assay. Crucially, alternative diagnoses, particularly MS and AQP4+NMOSD, must be excluded. Supporting features that enhance diagnostic confidence include longitudinal optic nerve involvement, perineural enhancement, and the “H-sign” on spinal MRI. CSF analysis typically reveals mild pleocytosis and elevated protein levels, while oligoclonal bands are present in only about 15% of cases, further distinguishing MOGAD from MS (Table 2).

### 3.4. Atypical Presentations in MOGAD: Expanding the Clinical Spectrum

Although MOGAD should be diagnosed considering the six core clinical syndromes, emerging evidence reveals a broader and more nuanced clinical spectrum. These atypical manifestations can mimic a range of neurological, rheumatological, and ophthalmological disorders, often leading to diagnostic uncertainty and delays.

#### 3.4.1. Overlap with Autoimmune Encephalitis

MOGAD may co-occur with other autoimmune encephalitides, particularly anti-NMDAR encephalitis. Patients—often children or young adults—may present with overlapping features such as optic neuritis alongside psychiatric symptoms, seizures, and dyskinesias. Anti-NMDAR antibodies may be detected concurrently or sequentially; less frequent associations have been reported with GFAP and CASRP2 antibodies [25,26,27,28,29]. The co-occurrence of MOGAD with other autoimmune encephalitides suggests potential shared or interacting immunopathological pathways. MOGAD may represent a broader predisposition to autoimmune responses in the CNS, where the initial trigger or ongoing inflammation can activate B-cells and T-cells that cross-react with other neuronal or glial targets. Also, an infection or environmental factor could trigger an immune response against MOG, and this response might inadvertently activate immune cells that target other brain antigens due to molecular similarities or a generalised inflammatory environment, similar to what has been proposed with herpes simplex 1 and NMDAR encephalitis. Also, the cytokine storm and immune cell infiltration observed in MOGAD, including increased Th2 and Th17 cells and elevated IL-6, GM-CSF, and IFN-γ, could create an environment conducive to the development of other autoimmune conditions or the exacerbation of pre-existing subclinical autoimmune processes. Therefore, comprehensive autoantibody panels are essential in complex neuroinflammatory syndromes, as the presence of one antibody does not exclude others.

#### 3.4.2. Atypical Ocular Inflammation

Beyond optic neuritis, MOG-IgG has been implicated in recurrent, steroid-responsive uveitis, scleritis, and episcleritis, and sometimes in isolation. Although uveitis has been commonly associated with MS, a 2016 study showed that uveitis was present in 2/5 of atypical MS patients presenting with positive MOG-IgG [30]; ocular manifestations have been increasingly reported in MOGAD [31,32,33,34]. The involvement of MOG-IgG in non-optic neuritis ocular inflammation suggests that MOG or similar antigens may be present or expressed in other ocular tissues, or that the immune response has systemic manifestations [35]. MOG-IgG testing should be considered in patients with idiopathic or refractory bilateral ocular inflammation, particularly when neurological symptoms are present.

#### 3.4.3. Intracranial Hypertension Syndromes

MOGAD can mimic or coexist with intracranial hypertension (IH), presenting with headaches, papilledema, and visual field deficits. These may occur with or without optic neuritis and are sometimes associated with meningeal enhancement on MRI. MOGAD’s association with IH is likely due to inflammation affecting intracranial pressure regulation [36,37,38,39]. A cross-sectional study investigated elevated intracranial pressure (ICP) in 41 AQP4+NMOSD and 37 MS patients. The findings indicated that 17% of NMOSD patients and 11% of MS patients presented with raised opening pressure. Notably, these patients were largely asymptomatic and younger than those with normal opening pressure. Additionally, obesity was more prevalent in the AQP4+NMOSD group with elevated ICP (57%) compared to the normal opening pressure group (12%) [40]. While the exact mechanisms are still being researched, MOG-IgG antibodies could trigger an inflammatory response in the arachnoid villi or cerebral veins, impairing CSF reabsorption and leading to increased intracranial pressure. This is supported by cases where meningeal enhancement is observed on MRI. Although MOG is primarily found on oligodendrocytes, its damage could indirectly contribute to CSF dynamics changes or alter glial-vascular unit function, influencing fluid homeostasis. Also, while AQP4 antibodies are specifically linked to NMOSD and water channel dysfunction, MOGAD’s inflammatory processes might still have some impact on fluid balance or the blood–brain barrier integrity, albeit through different mechanisms. MOGAD should be considered in atypical IH presentations, especially in non-obese patients or those unresponsive to standard treatments.

#### 3.4.4. Cranial Neuropathies

Involvement of cranial nerves beyond the optic nerve is increasingly recognised. Documented presentations include facial palsy (CN VII), trigeminal neuralgia (CN V), vestibulocochlear symptoms (CN VIII), and oculomotor palsies (CN III, IV, VI) [41,42,43,44]. This points to a broader inflammatory process that can affect both central and peripheral nervous system components of these nerves. Although cranial nerves are part of the peripheral nervous system, they have CNS components at the root entry zone (REZ) or tracts within the brainstem where MOG is present. MOG-IgG could target myelin in these regions, leading to demyelination and nerve dysfunction. Also, co-existence with other antibodies targeting peripheral myelin or cranial neuropathies secondary to meningeal inflammation could also be present in these cases. Inflammatory cranial neuropathies with corresponding brainstem lesions, particularly at the REZ, should prompt evaluation for MOGAD.

#### 3.4.5. Aseptic Meningitis and Hypertrophic Pachymeningitis

Recent research has identified meningitis-like presentations as a rare but increasingly recognised atypical phenotype of MOGAD. MOGAD has now been shown to occasionally manifest with clinical and radiological features resembling aseptic meningitis. In a multicenter study, meningitis was documented as an attack phenotype in MOGAD, with patients presenting with headache, fever, neck stiffness, and CSF pleocytosis, often without classic demyelinating lesions on initial imaging [45]. These episodes were sometimes misdiagnosed as infectious meningitis, delaying appropriate immunotherapy. Another study further characterised this presentation in a Chinese cohort, noting that meningitis-like attacks were frequently underrecognized and could precede or accompany typical MOGAD features. Importantly, these patients often responded well to corticosteroids, reinforcing the autoimmune nature of the syndrome [46]. Also, both focal and diffuse hypertrophic pachymeningitis have been reported in pediatric and adult-onset cases of MOGAD [47,48]. Pathological insights support this clinical observation, revealing inflammatory meningeal infiltration and perivascular demyelination in MOGAD cases, which may underlie the meningitis symptoms [22]. 

**Figure 1:**
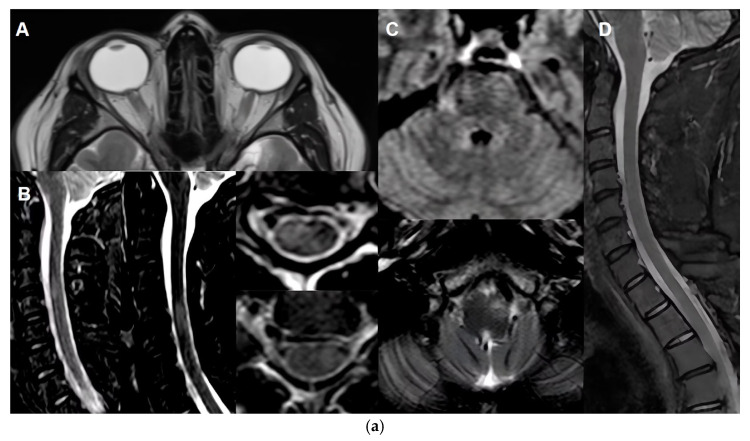

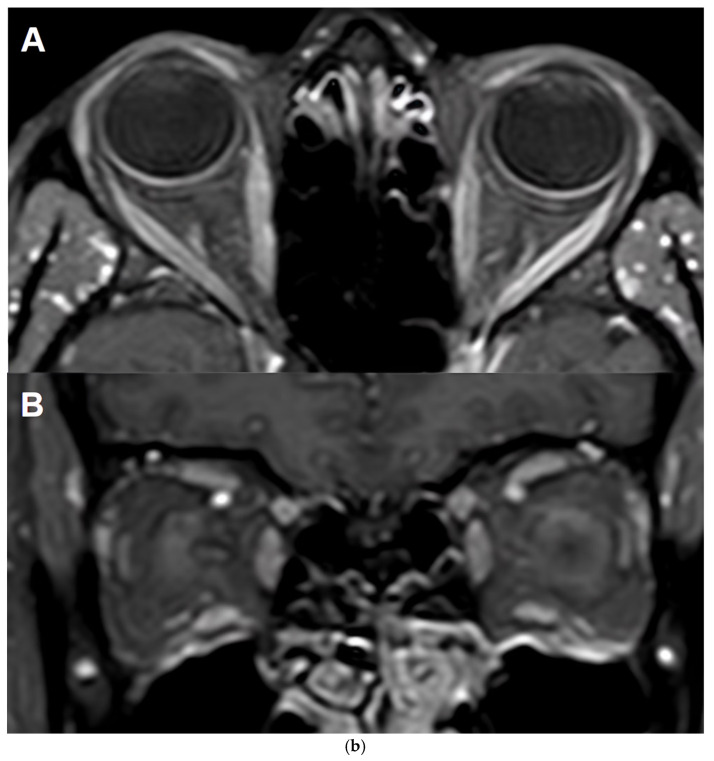
Atypical presentations in MOGAD. (**a**): Initial Presentation: 28 yo man. Prodromal headache with bilateral optic disc oedema. Cerebrospinal fluid (CSF) opening pressure: 24 cm. Disease Evolution: Developed bilateral optic neuritis, myelitis, and brainstem syndrome. First Relapse: One month later, a new brainstem relapse occurred. CSF opening pressure increased to 28 cm. Anti MOG IgG positive (without titre). Key MRI findings: (A) Bilateral optic disc oedema. (B) Multiple multisegmental demyelinating lesions affecting the spinal cord, showing signs of inflammatory activity. (C) Infratentorial T2 hyperintense lesions. (D) Resolution of spinal cord lesions. (**b**): Initial Presentation: 50 yo woman. Headache, pain around the left eye, followed by red eye and photophobia, visual field disturbance. Disease Evolution: Anti MOG IgG 1:100 (high titre). Resolves with topic steroids. Key MRI findings: (A) Signs of posterior episcleritis with (B) edematous changes in retrobulbar fat.

Together, these findings expand the clinical spectrum of MOGAD and underscore the need for heightened awareness among clinicians, especially when encountering patients with recurrent or steroid-responsive meningitis-like episodes. Early recognition of this atypical presentation is crucial to avoid misdiagnosis and ensure timely immunomodulatory treatment (Table 3 and Figure 1).

*Key takeaways for clinicians include:*

- Maintain diagnostic flexibility in inflammatory presentations that deviate from classical demyelinating patterns.
- Expand antibody testing in complex or seronegative neuroinflammatory syndromes.
- Recognise red flags such as recurrent scleritis, atypical intracranial hypertension, aseptic meningitis/hypertrophic pachymeningitis or unexplained cranial neuropathies.
- Further prospective studies are needed to clarify the prevalence, pathophysiology, and prognostic implications of these atypical manifestations.

## 4. Treatment Paradigms and Emerging Therapies

Management of MOGAD requires a nuanced approach that balances aggressive treatment of acute attacks with individualised long-term strategies to prevent relapses. Unlike MS or AQP4+NMOSD, MOGAD currently lacks universally approved disease-modifying therapies or standardised treatment protocols. Consequently, current recommendations primarily stem from observational data and the consensus of expert guidelines.

### 4.1. Acute Treatment

Management of acute attacks in MOGAD is centred on rapid immunosuppression to limit demyelination and promote recovery. First-line therapy includes intravenous methylprednisolone (IVMP) at a dose of 1 g/day for 3–5 days, ideally initiated within 24–72 h of symptom onset. Following IVMP, a prolonged oral corticosteroid taper over 2–3 months is recommended to reduce the risk of early relapse, typically starting at 1 mg/kg/day of prednisone. Clinical response is the primary measure of treatment efficacy, with repeated MRI reserved for atypical or refractory presentations [49,50,51,52,53,54].

In cases where patients are refractory to corticosteroids, escalation to plasma exchange (PLEX) or intravenous immunoglobulin (IVIG) is warranted. PLEX is typically administered in 5–7 sessions over 10–14 days, while IVIG is dosed at 2 g/kg over 2–5 days. In severe or steroid-unresponsive cases, combination therapy with PLEX and IVIG or the use of anti-IL-6 agents may be considered [49,50,51,52,53,54] (Table 4).

### 4.2. Maintenance Therapy

Long-term immunosuppressive therapy can be considered for patients with persistent MOG-IgG seropositivity beyond six months, severe initial attacks, a relapsing disease course, or high-risk features such as cortical encephalitis, female sex, or older age. Several off-label immunosuppressive agents are used in clinical practice, each with distinct mechanisms and monitoring requirements [49,50,51,52,53,54] (Table 5).

### 4.3. Ineffective or Harmful Therapies

Several disease-modifying therapies (DMTs) commonly used in MS have been shown to be ineffective or potentially harmful in MOGAD. Interferon-beta may exacerbate disease activity, while natalizumab has been associated with breakthrough relapses. Fingolimod has been linked to severe relapses and disease worsening. Therefore, MS-approved DMTs generally do not prevent relapses in MOGAD and are not recommended [50,51].

### 4.4. Treatment Considerations During Family Planning and Pregnancy

Patients with family planning and pregnancy present unique clinical challenges. While relapse risk tends to decrease during pregnancy—particularly in the third trimester—it significantly increases in the postpartum period, especially within the first three months. This pattern underscores the importance of preventive strategies during high-risk windows. Corticosteroids are generally considered safe (risk of cleft palate, especially in the first trimester, OR 1.0 95%CI (0.7–1.4) [55] and effective for managing acute attacks during pregnancy, while IVIG is a suitable option for both pregnancy and lactation. A multidisciplinary approach is essential, beginning with pre-conception counselling as some of the chronic immunosuppression therapies might be teratogenic and require a wash-out period or change in maintenance therapy, continuing with trimester-specific treatment planning and close monitoring throughout pregnancy and postpartum. Breastfeeding may offer a modest protective effect, but individualised care remains critical to balance maternal health and fetal safety [56,57,58,59,60].

### 4.5. Treatment Discontinuation Considerations

Decisions regarding the discontinuation of immunosuppressive therapy (IST) in MOGAD must be approached cautiously. A Korean cohort study found that 24.4% of patients experienced a relapse after stopping IST. Notably, all relapses occurred in patients with a prior relapsing course, while none of the patients with a monophasic history relapsed. The study also revealed that shorter IST duration (<12 months) was significantly associated with a higher risk of relapse compared to longer treatment durations [61]. Based on these findings, a minimum of two years of relapse-free follow-up could be recommended before considering treatment withdrawal. Even then, close clinical and radiological monitoring is essential for at least 12–24 months post-discontinuation to detect early signs of recurrence.

### 4.6. Emerging Therapies and Clinical Trials

The therapeutic landscape for MOGAD is being actively shaped by two landmark Phase 3 clinical trials aimed at establishing the first evidence-based, targeted treatments for relapse prevention. The COSMOG trial (NCT05063162) is a randomised, double-blind, placebo-controlled study evaluating rozanolixizumab, a neonatal Fc receptor (FcRn) inhibitor [62]. By blocking FcRn, the therapy aims to accelerate the degradation of pathogenic MOG-IgG autoantibodies. In parallel, the METEREOID trial (NCT05271409) is a similarly designed study investigating satralizumab, a monoclonal antibody that targets the interleukin-6 (IL-6) receptor to inhibit a key inflammatory pathway implicated in MOGAD pathogenesis [63]. Both trials share the primary endpoint of measuring the time to a patient’s first relapse and assess critical secondary outcomes, including the annualised relapse rate (ARR) and changes in disability as measured by the Expanded Disability Status Scale (EDSS). The results of these studies are highly anticipated and expected to define the future standard of care.

Beyond antibody and cytokine-targeted therapies, a new frontier is emerging with the investigation of Chimeric Antigen Receptor (CAR) T-cell therapy for severe, refractory MOGAD. This innovative approach, which involves genetically engineering a patient’s T-cells to eliminate antibody-producing B-cells, has shown initial promise. A published case report detailed successful treatment of a refractory MOGAD patient with CD19-directed CAR T-cells, which resulted in B-cell depletion and a reduction in MOG-IgG titers [64]. Building on this concept, research is advancing with more targeted approaches. For instance, two Phase 1 open-label studies are now underway to evaluate the safety and efficacy of in relapsing/refractory autoimmune conditions, including MOGAD (NCT04561557 CT103A and NCT06869278 LCAR-AIO CAR-T) [65,66]. These trials represent a potential paradigm shift toward a highly specific cellular therapy that could offer deep and durable remission, though further data is required to establish its role.

## 5. Predicting Prognosis, Relapse Risk, Adapting and Monitoring

Understanding the long-term trajectory of MOGAD is essential for guiding treatment decisions and patient counselling. While many individuals experience a monophasic course with favourable recovery, a significant proportion develop relapsing disease, which can lead to cumulative neurological disability. Relapsing disease course in MOGAD is a major determinant of long-term disability, making early identification of individuals at risk crucial for guiding treatment. Several clinical, serological, and immunological features have been associated with an increased risk of relapse. Predicting who will relapse, how frequently, and with what severity remains a central challenge in clinical practice. This section explores the evolving evidence on disease prognosis, introduces tools for relapse risk stratification, and highlights emerging strategies for biomarker and imaging-based monitoring.

### 5.1. Clinical Risk Factors

The historic knowledge showed that approximately 30–50% of patients follow a monophasic course, with no further disease activity after the initial episode [8]. Longer follow-up periods reveal a higher incidence of relapsing courses in MOGAD. A comprehensive review of 4699 MOGAD patients indicates that 35% experience a relapse within the first year, 50% by five years, and as many as 70% by ten years [3]. This trend underscores how shorter follow-up durations in earlier studies contributed to underreported relapse frequencies [1,3,8,21].

#### 5.1.1. Age, Sex and Phenotype at Onset

Age, sex, and initial clinical presentation are key prognostic factors in MOGAD, influencing both relapse risk and long-term disability. While MOGAD affects individuals across the lifespan, current evidence suggests that adults are more likely to experience a relapsing disease course [3,21,67]. In children, patients presenting with ON have a higher risk of relapse compared to ADEM at onset. In adults, ON is also associated with a higher risk of relapse compared to TM, particularly in younger adults <40 years old [64,65,66,67,68]. At the other end of the spectrum, late-onset MOGAD (onset after age 50) is linked to greater disability accumulation, often due to diagnostic delays and the presence of age-related comorbidities that obscure the clinical picture or complicate treatment decisions [23,24]. Additionally, male sex has been associated with a reduced risk of relapse, suggesting a possible sex-related immunological influence on disease course [69].

While TM at onset is associated with a lower relapse risk, it tends to result in greater long-term disability, likely due to the severity of spinal cord involvement. These findings underscore the importance of early phenotypic characterisation in guiding treatment decisions and long-term monitoring strategies [64,65,66,67,68,69,70,71].

#### 5.1.2. Time to Acute Treatment, Prednisone Taper Duration and Early Maintenance Therapy

Acute treatment (<7 days from disease onset) and corticosteroid treatment for at least 1 month (preferably >5–12 weeks) have been associated with a lower risk of relapse in several studies [3,67,68,69,72,73,74]. Early maintenance therapy has also been associated with lower relapse risk [68,70,73], even in patients who discontinue treatment [61].

These correlations between early, acute treatment and the risk of relapse, even following the discontinuation of long-term maintenance therapy, suggest a distinct immunological and pathophysiological foundation for MOGAD. This differentiates it from previously characterized MS and AQP4+NMOSD, emphasizing the importance of prompt diagnosis and opportune interventions to avert subsequent relapses.

### 5.2. Predicting Relapse: Gut Feeling Versus Scores and Nomograms

Recent studies have developed predictive models aimed at stratifying the risk of relapse in patients with MOGAD. These tools represent a significant effort to move towards more personalized clinical management by identifying patients who may benefit most from early and ongoing immunotherapy [75,76].

The first study, a single-center analysis developed a nomogram to predict the risk of relapse within one year of disease onset. The study included 88 MOGAD patients from the First Hospital of Shanxi Medical University. Through logistic regression analysis, four independent risk factors for a one-year relapse were identified: female sex, a clinical phenotype of cortical encephalitis (CCE), a serum MOG antibody titer of ≥1:32, and receiving inadequate maintenance therapy after the first attack. The resulting nomogram demonstrated strong predictive performance, with an area under the curve (AUC) of 0.866 in the training cohort and 0.864 in the validation cohort. The model effectively stratified patients into low-risk and high-risk groups, with a 1-year relapse rate of 18% in the low-risk group versus 61% in the high-risk group. The study also noted the model showed value in predicting relapse at two years (AUC of 0.817) [75].

The second study, utilized a larger, multicenter cohort from the China National Registry of Neuro-Inflammatory Diseases (CNRID) to develop and validate a simpler scoring system, the MOG-AR score. This study included 188 patients and 612 treatment episodes. The MOG-AR score incorporates five factors: lack of Immunosuppressive therapy (5 points), use of oral Corticosteroids for less than 3 months (3 points), Onset Age of 45 years or older (2 points), female Sex (2 points), and the initial Attack phenotype (CCE 4 points, ADEM 3 points, optic neuritis 2 points, cerebral monofocal or polyfocal deficits 1 point, brainstem or cerebellar deficits 1 point, myelitis 1 point). A MOG-AR score of 9 or higher was found to be predictive of relapse (HR: 2.66), and patients with a score of 13-16 had a 78.8% risk of relapse (HR 3.29), compared to 33.3% for those with a score of 0-4. While practical, the overall predictive ability of the MOG-AR score had an AUC of 0.7091515 [76]. Authors suggest that a score of 9 or higher is considered predictive of relapse and may guide decisions regarding the initiation or continuation of maintenance immunotherapy. This tool is particularly useful in clinical practice to personalise long-term treatment strategies [76] (Table 6).

Although these scores are a promising step forward, their immediate application in global clinical practice should be approached with caution. The most significant limitation of both studies is that they were developed and validated exclusively within Chinese patient cohorts. The risk factors for MOGAD relapse may differ across various ethnic and geographic populations due to genetic, environmental, or healthcare system differences. Therefore, the generalizability of these models’ other diverse patient groups is unknown.

### 5.3. Biomarker and Imaging Monitoring

Monitoring disease activity in MOGAD is an evolving field, with several promising tools under investigation. While imaging modalities such as MRI and optical coherence tomography (OCT) are valuable for assessing structural damage, the role of serum MOG-IgG titers as a longitudinal biomarker is still under active investigation and debate.

#### 5.3.1. Longitudinal Assessment of MOG-IgG Titers

The utility of serial MOG-IgG titers in predicting relapses is controversial [77,78,79]. Several studies have shown that MOG-IgG titers at disease onset do not reliably differentiate between monophasic and relapsing courses. For instance, a pediatric cohort of 116 children with long-term follow-up demonstrated similar onset titers in both groups (relapsing and monophasic), suggesting limited prognostic value at baseline [80]. However, the same study also reported that a decrease in titre, particularly below the 1:160 threshold during the first 24 months of follow-up, had a high positive predictive value for a monophasic course.

Other studies also support the potential role of follow-up titers—particularly those measured during remission—in risk stratification [81,82,83]. A multicenter Italian study with pediatric and adult onset patients found that remission titers >1:2560 were significantly associated with a relapsing course (HR: 10.9, *p* < 0.001). Moreover, relapses were rarely observed in patients with titers below 1:40, and seroconversion to negativity was associated with a 95% reduction in relapse incidence. These findings suggest that high remission titers may serve as a red flag for future disease activity, especially when integrated with clinical and radiological data [83].

Despite these findings, other studies caution against over-reliance on MOG-IgG titers. Persistent seropositivity does not guarantee relapse, and relapses can occur even after seroreversion [21,80,82]. Furthermore, titers may fluctuate due to immunotherapy or sampling timing, and inter-laboratory variability in assay methods limits the comparability of results. The core of the disagreement stems from the lack of assay standardization, particularly between the more accurate live cell-based assays (CBAs) and the more widely available, but less reliable, fixed CBAs. Moreover, the term “seroreversion” often reflects titers falling below assay thresholds rather than complete antibody clearance, which complicates the interpretation of longitudinal titers and reduces their standalone clinical utility.

Given the current evidence, routine longitudinal measurement of MOG-IgG titers could be recommended for selective follow-up testing—particularly at 6 to 12 months post-onset—and may be informative in specific clinical contexts, such as in pediatric patients or those with high-risk features. Persistent high titers during remission may support the decision to continue immunosuppressive therapy, while seroconversion may inform de-escalation strategies. Clinical context, disease severity, and imaging findings must be integrated into any decision to withdraw immunotherapy. Further prospective studies are needed to validate reliable biomarkers for safe treatment discontinuation.

Beyond the utility of persistence of MOG-IgG antibodies, specific epitope recognition patterns are being investigated for prognostic value. Recent insights suggest that the pattern of MOG epitope recognition by serum MOG-IgG may predict disease course. Patients whose antibodies target non-P42 epitopes—distinct from the canonical P42 site—are more likely to experience a relapsing disease course, especially in pediatric and young adult populations. This epitope-specific profile appears to be stable over time and may serve as an early biomarker for relapse risk stratification. This association is particularly strengthened when combined with unilateral optic neuritis at onset, where non-P42 MOG-IgG was associated with more than double the risk of relapse in adult patients [80,84]. In pediatric onset patients, non-P42 patients presented with earlier median age at onset, higher frequency of ADEM, and a higher relapse rate [85]. These findings might support the integration of epitope-specific testing into routine MOGAD diagnostics to inform prognosis and guide individualized treatment strategies. Further research is needed to solidify their utility as a definitive biomarker.

Ultimately, treatment decisions should be guided by a combination of clinical history, imaging findings, and biomarker trends rather than serology alone. Further prospective studies are needed to validate the predictive value of MOG-IgG kinetics and to define standardised thresholds for clinical use.

#### 5.3.2. MRI Monitoring in MOGAD

MRI plays a critical role in the diagnosis, differential diagnosis and monitoring of MOGAD [1,86], particularly in assessing optic nerve, spinal cord, and brain involvement. However, its utility in long-term monitoring is nuanced due to the disease’s unique radiological behaviour.

*Resolution of Lesions*

MOGAD lesions, especially in the spinal cord and brain, tend to resolve more completely than in other demyelinating disorders. Studies report that over 70% of brain and spinal cord lesions show complete resolution on follow-up MRI [1,87,88,89]. Unlike AQP4+NMOSD, MOGAD rarely leads to spinal cord atrophy or persistent T1 hypointensity, although localised gray matter volume reduction may occur in previously affected areas. A study provided compelling evidence supporting the diagnostic utility of MRI in distinguishing MOGAD from MS through longitudinal imaging. By analyzing paired MRI scans during clinical attacks and remission, the study found that resolution of T2-hyperintense lesions was significantly more common in MOGAD than in MS. Specifically, resolution of at least one T2 lesion had a sensitivity of 77–100% and specificity of 86–99% for identifying MOGAD, while resolution of two or more lesions was 100% specific. Additionally, MOGAD patients were more likely to have completely normal follow-up MRIs—32% in the brain and 78% in the spinal cord—compared to none in the MS cohort [90]. These findings suggest that lesion resolution, particularly at one-year follow-up, may serve as a valuable biomarker for MOGAD diagnosis and could help establish a new imaging baseline for long-term monitoring.

*Asymptomatic (Silent) Lesions*

Silent lesions—those not associated with clinical symptoms—are rare in MOGAD, particularly during remission. One retrospective study including 182 patients with MOGAD found that only 3% of remission MRIs revealed new silent lesions, and these were often predictive of imminent relapse, with a median time to relapse of 2 months [91]. In the same study, silent lesions detected during attacks were more frequent, presenting in about 3% of attack MRIs. Another retrospective study including 203 patients reported 13% of interattack MRIs showing optic nerve enhancement, mostly at prior sites of ON (81%), in the context of ADEM without a concomitant eye exam (14%) or with prior history of ON but not concomitant MRI (5%) [92]. These findings suggest that while silent lesions are uncommon, their presence may warrant closer clinical attention.

*Recommendations for MRI Monitoring and Follow-Up*

The French NOMADMUS Expert Group [93] recommends a structured MRI monitoring protocol for MOGAD:

- At Diagnosis: Perform MRI of the brain, optic nerves, and spinal cord, with and without gadolinium, ideally on a 3T scanner for brain and optic nerves and ≥1.5T for spinal cord.
- Post-Relapse (6 Months): Repeat MRI centered on the affected area (brain, optic nerves, or spinal cord), with and without gadolinium, to assess lesion resolution and rule out persistent enhancement.
- Long-Term Monitoring (Every 36 Months): In the absence of clinical activity, perform gadolinium-free MRI of the brain (±optic nerves) and spinal cord to ensure radiological stability and monitor for treatment-related complications.

These intervals balance the low frequency of silent lesions with the need for safety surveillance, especially in patients on long-term immunosuppressive therapies.

#### 5.3.3. Optical Coherence Tomography (OCT) in MOGAD

OCT is a valuable, non-invasive tool for tracking retinal nerve fibre layer (RNFL) thickness, especially in patients with optic neuritis-dominant phenotypes. It helps detect subclinical damage and monitor recovery. It provides a non-invasive, high-resolution assessment of retinal structure, particularly useful in patients with ON, the most common manifestation of MOGAD [1].

OCT can quantify thinning of the peripapillary RNFL (pRNFL) and macular ganglion cell-inner plexiform layer (mGCIPL), which correlate with visual function and cumulative axonal damage [94,95,96].

In MOGAD, ON is often bilateral and anterior compared to MS and AQP4+NMOSD, leading to pronounced acute retinal oedema followed by neuroaxonal degeneration. Unlike AQP4+NMOSD, where ON episodes are less frequent but more destructive, MOGAD patients tend to experience more frequent ON episodes with relatively better visual recovery [94,97,98]. However, repeated attacks can lead to progressive retinal thinning and visual impairment. OCT is also being investigated for its potential to detect subclinical damage and differentiate MOGAD from MS and AQP4+NMOSD based on retinal layer patterns [99,100,101]. Its accessibility and reproducibility make it a valuable tool for longitudinal monitoring in both adult and pediatric populations (Table 7).

#### 5.3.4. Beyond “Relapse-Free” MOGAD

Understanding the patient experience in demyelinating disorders such as MOGAD is essential for advancing care beyond traditional clinical endpoints. While treatment efficacy and safety remain foundational in evaluating therapeutic interventions, they often fail to capture the full spectrum of disease burden as experienced by patients. Patient-reported outcomes (PROs) offer a vital lens into the real-world impact of symptoms on daily functioning, emotional well-being, and social participation. In conditions like MOGAD—characterized by heterogeneous and often fluctuating neurological symptoms—PROs help illuminate the nuanced challenges patients face, such as chronic pain, fatigue, and cognitive difficulties, which may persist even in the absence of clinical relapse. Incorporating PROs into clinical trials and routine clinical care models ensures that therapeutic strategies are aligned with what truly matters to patients, fostering more holistic, patient-centered approaches to disease management.

In this context, studies have tried to address the impact of MOGAD on several quality of life outcomes, highlighting, for instance, significantly worse scores in anxiety, stigma, cognitive function and social interaction [102] with even worse psychological quality of life of MOGAD patients compared to AQP4+NMOSD [103].

A recent qualitative study explored the lived experience of individuals with MOGAD through in-depth interviews with patients and clinicians. The study developed a comprehensive conceptual model capturing 32 symptoms across seven domains and 50 health-related quality of life (HRQoL) impacts across eight domains. Patients most frequently reported symptoms such as eye pain, fatigue, body aches, headaches, and blurred vision, alongside significant HRQoL challenges including difficulty with daily activities, inability to work, depression, and impaired mobility [104]. These findings underscore the importance of integrating patient perspectives into clinical research and highlight the need for future therapeutic trials to address not only disease control but also the broader, deeply personal impacts of MOGAD on patients’ lives.

## 6. Regional and Global Perspectives

The clinical understanding and management of MOGAD continue to evolve across diverse healthcare settings worldwide. While the disease exhibits a relatively consistent immunopathological profile, regional differences in diagnostic access, treatment availability, and patient demographics significantly influence outcomes. Variability in serological testing protocols, imaging resources, and therapeutic strategies highlights the need for harmonised global guidelines. This section explores how MOGAD is recognised and managed across different regions, emphasising disparities, emerging collaborative efforts, and the importance of inclusive research to ensure equitable care for all patients.

### 6.1. Latin America: Emerging Data, Diversity and Unique Challenges

Latin America has become an increasingly important contributor to the global understanding of MOGAD, offering insights shaped by its unique demographic diversity, healthcare infrastructure, and clinical realities.

Ethnic and racial diversity in Latin America has also provided a rich context for exploring immunological and clinical variability in MOGAD. This diversity includes different prevalences among pediatric and adult-onset cases between countries or even cities, and also in the proportion of MOG-IgG positive in patients with seronegative NMOSD. [105,106,107,108].

A multicenter study revealed that clinical outcomes in optic neuritis did not vary across Latin American ethnic groups [109], while in a study based in the USA, Hispanic/Latino ethnicity was associated with a higher risk of relapsing MOGAD [70]. These findings underscore the importance of inclusive research encompassing other relevant factors, such as social determinants of health when exploring the interaction between race/ethnicity and clinical outcomes.

Latin American researchers have also evaluated the real-world applicability of international diagnostic criteria. In a 2024 study, a multicenter study assessed the 2023 international MOGAD criteria in a Latin American cohort, finding high diagnostic concordance but identifying implementation challenges in resource-limited settings. Encouragingly, the 2023 MOGAD diagnostic criteria demonstrated high sensitivity (86%) and specificity (100%) in this LATAM cohort, validating their use in real-world settings, even in the face of limited access to MOG-IgG titration [110]. National and regional registries have provided valuable longitudinal data on attack frequency, treatment response, and relapse risk, offering a counterpoint to European and North American datasets.

A major milestone in this effort is the Latin American RAND/UCLA modified Delphi consensus published in 2025, which brought together experts from 13 countries to develop region-specific recommendations for MOGAD management [111]. These guidelines emphasize early antibody testing, individualized immunotherapy, and pragmatic follow-up strategies, tailored to the constraints of local healthcare systems. Unlike European and North American protocols, which often assume access to advanced imaging and monoclonal antibodies, the Latin American consensus prioritizes feasibility and equity, reflecting the realities of public health systems where diagnostic delays and treatment gaps are common.

Treatment patterns in the region reflect both clinical need and resource availability. Corticosteroids were used in 95% of acute attacks, while rituximab emerged as the most common maintenance therapy (53.2%), followed by azathioprine (24.6%) and mycophenolate mofetil (13.9%). These findings underscore the importance of practical, accessible treatment strategies in LATAM. The 2025 Latin American consensus recommendations reinforced this approach, advocating for early initiation of high-dose intravenous methylprednisolone (1 g/day for 3–5 days), followed by a 3–6 month oral prednisone taper. In steroid-refractory cases, plasma exchange (PLEX) and IVIG were recommended, with early PLEX initiation (within 20 days) linked to better outcomes. For long-term management, the consensus emphasised the use of cost-effective immunosuppressants like azathioprine and mycophenolate, while also recognising the role of rituximab and IVIG in selected patients. Importantly, MS-specific disease-modifying therapies such as interferon-beta, fingolimod, and natalizumab were discouraged due to a lack of efficacy and potential harm in MOGAD [111,112].

Despite these advances, Latin American cohorts continue to face systemic barriers that differentiate their experience from patients in high-resource settings. Access to MOG-IgG testing remains uneven, with many centres relying on international laboratories or limited local capacity. MRI monitoring, though recognized as essential, is often constrained by equipment availability, scheduling delays, and lack of standardized protocols. Treatment access is similarly variable; while corticosteroids and azathioprine are widely used, newer biologics such as rituximab, tocilizumab, and satralizumab may be unavailable or unaffordable for many patients. These disparities not only affect clinical outcomes but also limit the ability to conduct multicenter trials and apply evidence-based guidelines. As Latin American researchers continue to contribute meaningfully to the global MOGAD literature, there is an urgent need for investment in diagnostic infrastructure, training, and equitable access to therapies. Bridging these gaps will be essential to ensure that the insights generated in the region translate into improved care for all patients living with MOGAD.

### 6.2. The Chilean Cohort: A National Perspective

The Chilean experience with MOGAD provides a valuable lens into the disease’s clinical heterogeneity, diagnostic challenges, and treatment disparities. Drawing from multicenter longitudinal registry data, case series, and neuro-ophthalmological studies, the Chilean cohort offers a nuanced understanding of MOGAD in both pediatric and adult populations.

*Demographics and Clinical Spectrum*

The national cohort includes 95 patients, with a female-to-male ratio of 2:1 and a median age at onset of 34 years. Pediatric cases account for 20% of the population, reflecting the disease’s bimodal age distribution. A relapsing disease course was observed in 40% of patients, with relapsing individuals being younger at onset (median 24 years vs. 33 years in monophasic cases) and having significantly longer disease duration (median 63.5 vs. 6 months, *p* = 0.004). The most common initial presentations were optic neuritis (34%) and myelitis (22%), with pediatric patients more frequently presenting with encephalitis or ADEM-like syndromes (75%) [113,114].

*Neuro-Ophthalmological Features*

A sub-study of 40 MOGAD patients with ON revealed that ocular pain (58%) and optic disc swelling (48%) were common. MRI and OCT findings were consistent with international MOGAD-ON patterns, including perineuritis and anterior optic nerve involvement. Interestingly, 60% of patients reported retro-orbital headache preceding visual symptoms, suggesting a potential early clinical clue. Despite severe initial visual loss, long-term outcomes were generally favourable, with a median LogMAR VA of 0.1 (20/25 Snellen equivalent) and EDSS of 1.0 at last follow-up. Nonetheless, follow-up OCT showed severe pRNFL atrophy (median 73 (range 44–95) in affected eyes [115].

*Treatment Approach and Outcomes*

Acute treatment was widely accessible, with 89% receiving IV methylprednisolone and 75% oral steroids. Plasma exchange (5%) and IVIG (11%) were used less frequently. Chronic immunosuppression was initiated in 71% of patients, with mycophenolate mofetil (47%), azathioprine (22%), and rituximab (20%) being the most commonly used agents. Notably, 20% of relapses occurred under chronic therapy, prompting treatment escalation in some cases, including the use of satralizumab or monthly IVIG [113,114].

Disability outcomes were generally favourable, with a median EDSS of 1.5 in relapsing patients and 0 in monophasic cases. However, patients in the public healthcare system experienced significantly longer diagnostic delays (median 13 months) compared to those in the private healthcare system (median 1 month, *p* = 0.04), underscoring systemic disparities in access to diagnostics and care [113,114].

*Atypical Presentations and Co-positivity*

Approximately 7% of patients presented with atypical or non-core syndromes, including intracranial hypertension, sclerouveitis, anti-NMDAR autoimmune encephalitis, and multiple cranial neuropathies. These cases were often aggressive, with high CSF pleocytosis and prolonged hospitalisations, but responded well to combined immunotherapy. Three patients were co-positive for anti-NMDAR antibodies, presenting with cortical encephalitis and seizures—two pediatric patients with a FLAMES-like syndrome and excellent steroid response, and another adult patient with refractory status epilepticus, orofacial dyskinesia and poor visual recovery from concomitant bilateral optic neuritis. One patient exhibited both seropositive AQP4-IgG and MOG-IgG, though titers were unavailable. Given the patient’s presentation with typical Area Postrema Syndrome and a pathognomonic MRI lesion, false-positive MOG-IgG was considered, with a final diagnosis of AQP4+NMOSD [113,114].

*Diagnostic and Monitoring Challenges in Routine Clinical Practice*

While the 2023 MOGAD diagnostic criteria demonstrated excellent performance (100% sensitivity, 99.7% specificity) in this cohort, longitudinal MOG-IgG monitoring is limited due to out-of-pocket costs and availability. Only 8 patients had serial antibody testing, with 5 showing persistent positivity. MRI and OCT were used for monitoring, but access varied by region and healthcare system [113,114].

### 6.3. Global Initiatives and Collaborative Networks

A pivotal development in the study of MOGAD has been the recent formalisation of global research collaborations. These initiatives have been instrumental in moving the field from a collection of case series to a more unified clinical and scientific discipline. The most significant achievement of this global cooperation is the establishment of the International MOGAD Panel. This consortium, comprising a geographically and professionally diverse group of experts from North America, Europe, Asia, Australia, and South America, published its proposed diagnostic criteria for MOGAD in 2023. This landmark publication represents a critical inflexion point, providing the first internationally endorsed framework for diagnosis, which in turn standardises patient identification for both clinical practice and research endeavours, including therapeutic trials [1].

Along with the establishment of this panel, several patient advocacy organisations have played a crucial role in promoting research, raising awareness, and fostering a sense of community among MOGAD patients and their families. Organisations such as The MOG Project, the Siegel Rare Neuroimmune Association (SRNA), The Sumaira Foundation, and The Guthy-Jackson Charitable Foundation (GJCF) have been instrumental in fundraising, disseminating information, and connecting patients with resources. These efforts complement the academic and clinical collaborations, providing a crucial support system for patients navigating a rare and often challenging diagnosis. These global patient organisations also facilitate the collection of real-world data, contributing valuable insights into disease trajectories, treatment responses, and unmet needs, which often inform ongoing research and clinical trial design.

Also, regional networks have played a critical role in validating the criteria and exploring population-specific nuances. In Europe, the MOGAD Eugene Devic European Network (MEDEN) has spearheaded multicenter studies and fostered early-career researchers through dedicated fellowships. In Latin America, the Latin American MOGAD Network has emerged as a key collaborative platform, while in the Asia-Pacific region, groups such as the Australian and New Zealand MOGAD Study Group and Japanese consortia have contributed essential data on regional phenotypic and therapeutic variability.

Looking ahead, these global initiatives are crucial for defining best practices, developing standardised outcome measures, and conducting multicenter clinical trials that are essential for gaining regulatory approvals for new therapies. They also play a vital role in ensuring that research findings and clinical advances are disseminated widely, especially to regions with limited resources, fostering a more equitable landscape for MOGAD patients worldwide.

### 6.4. Future Directions in Global MOGAD Care

The global landscape of MOGAD management is rapidly evolving, driven by ongoing research, collaborative initiatives, and a deeper understanding of regional challenges. Future directions must focus on bridging existing gaps in diagnosis, access to care, and personalised treatment strategies.

*Key areas for improvement include:*

- Harmonisation of Diagnostic Practices: Despite the 2023 consensus criteria, variability in MOG-IgG testing methods (e.g., live vs. fixed cell-based assays, titre reporting) and interpretation persists. Standardising laboratory protocols and establishing international quality control programs are essential to ensure consistent and accurate diagnoses globally. This is particularly critical in resource-limited settings where access to advanced testing may be delayed or unavailable.
- Equitable Access to Therapies: Significant disparities exist in the availability and affordability of maintenance immunosuppressive therapies. Advocacy efforts are needed to ensure that effective treatments—both established and emerging—are accessible in all regions. This may involve exploring cost-effective generic options, implementing national MOGAD guidelines, and promoting healthcare policies that prioritise equitable access.
- Personalised Treatment Algorithms: As new therapies emerge from clinical trials, developing personalised treatment algorithms based on individual patient characteristics (age, clinical phenotype, relapse history, MOG-IgG kinetics) will be crucial. This approach moves beyond empirical treatment to evidence-based, tailored interventions that maximise efficacy while minimising side effects.
- Expansion of Global Registries and Biobanks: Expanding comprehensive, multinational patient registries and biobanks is vital for collecting real-world data, identifying novel biomarkers, and facilitating genetic and environmental research. These resources can help clarify the natural history of MOGAD, assess long-term treatment outcomes, and address knowledge gaps in underrepresented populations.
- Education and Awareness: Raising awareness among healthcare professionals and the public is paramount, particularly in regions where MOGAD is still underdiagnosed or misdiagnosed as MS. Educational initiatives can improve early recognition, reduce diagnostic delays, and ensure timely referral to specialised care.

By addressing these areas, the global MOGAD community can continue to advance personalised care, improve patient outcomes, and reduce the burden of this challenging demyelinating disorder worldwide.

## 7. Limitations

While this review offers a comprehensive synthesis of current knowledge on MOGAD, several limitations must be acknowledged to contextualise the findings and guide future research. First, the evidence base for MOGAD remains in evolution. Much of the current understanding of its pathogenesis, biomarker utility, and treatment strategies is derived from observational studies, expert consensus, and early-phase clinical trials. Robust longitudinal data and randomised controlled trials—particularly for maintenance therapies and emerging biologics—are still limited. This constrains the ability to draw definitive conclusions about long-term outcomes and optimal therapeutic approaches.

Second, although this review highlights data from Latin America, including a detailed analysis of the Chilean cohort, there remains a paucity of information from other underrepresented regions. Differences in healthcare infrastructure, diagnostic access, and treatment availability may limit the generalizability of these findings to broader global populations. Regional disparities also underscore the need for context-specific guidelines and resource-sensitive treatment algorithms.

Third, heterogeneity in diagnostic testing presents a significant challenge. Variability in MOG-IgG assay platforms—particularly between live and fixed cell-based assays—and the lack of standardised titer reporting can affect diagnostic accuracy and complicate relapse risk stratification across centres. This diagnostic variability may influence both clinical decision-making and the comparability of research outcomes. Furthermore, the application of other body fluid biomarkers, such as serum neurofilament light chain and glial fibrillary acidic protein, among others, is presently under investigation in research environments, with restricted clinical implementation, thus rendering their analysis beyond the scope of this review [116,117,118,119,120].

Fourth, family planning and pregnancy are increasingly recognised as relevant subjects of research; dedicated studies in these populations, particularly the latter, remain scarce.

Fifth, this review does not cover potential treatment strategies that remain in the preclinical stage. These emerging approaches, though promising, fall outside the scope of this article and warrant dedicated exploration in a separate review focused on experimental and early-phase interventions.

These limitations highlight the urgent need for continued international collaboration, standardised diagnostic protocols, and prospective multicenter studies. Such efforts will be essential to refine personalised care strategies and ensure equitable access to high-quality MOGAD diagnosis and treatment worldwide.

## 8. Conclusions

MOGAD has transitioned from a diagnostic enigma to a clearly defined autoimmune demyelinating disease, thanks to advances in immunopathology, imaging, and international consensus criteria. Its diverse clinical spectrum demands a nuanced, age-sensitive approach to diagnosis and care. The integration of tools like prognostic scores and emerging imaging and serum biomarkers is reshaping how clinicians monitor disease activity and tailor long-term therapy. Yet, as highlighted by Latin American cohorts, disparities in diagnostic access and treatment availability persist, underscoring the need for regionally adapted guidelines. Moving forward, global collaboration will be key to bridging these gaps, enabling personalised, biomarker-driven care that improves outcomes for MOGAD patients worldwide.

*Takeaway Messages:*

- Distinct Pathogenesis: MOGAD is primarily an antibody-mediated demyelinating disease targeting oligodendrocytes, distinct from MS and AQP4+NMOSD.
- Broad Clinical Spectrum: It presents with a wide range of neurological manifestations, from optic neuritis and myelitis to ADEM and cortical encephalitis, with age-dependent variability.
- Standardised Diagnosis: The 2023 international consensus criteria have greatly improved diagnostic accuracy, emphasising MOG-IgG seropositivity by cell-based assay and exclusion of mimics.
- Nuanced Treatment: Acute attacks respond well to corticosteroids, PLEX, and IVIG. Maintenance therapy is crucial for relapsing forms, including off-label options such as mycophenolate, azathioprine, rituximab and tocilizumab, while MS-specific DMTs are generally ineffective or harmful.
- Prognosis and Monitoring: While prognosis is generally good, a significant proportion of patient relapses. Tools like the MOG-AR score help stratify risk, and emerging biomarkers (NfL, GFAP) and imaging (OCT, MRI) aid monitoring. Longitudinal MOG-IgG titers remain debated for routine use, but persistent positivity may guide treatment.
- Global Disparities and Collaborations: Regional data highlight diagnostic delays and access inequities, underscoring the need for global collaboration, harmonised guidelines, and equitable access to care.

## Figures and Tables

**Table 1:** Clinical Phenotypes of MOGAD Across Age Groups.

Phenotype	Pediatric MOGAD [1,2,3,20,21,22]	Adult MOGAD [1,2,3]	Late-Onset MOGAD (>50 yrs) [3,23,24]
**Optic Neuritis (ON)**	Common, often bilateral, good visual recovery	Most frequent presentation (50–60%)	Most frequently at onset, often misdiagnosed
**Transverse Myelitis (TM)**	Less common, may follow ADEM	30–40% of cases, longitudinally extensive	May be confused with ischemic or degenerative causes
**ADEM**	Most common (40–50%), multifocal, encephalopathy	Rare in adults	Rare but cerebral symptoms may mimic ADEM
**Cortical Encephalitis**	Seizures in up to 20%	10–15% with seizures and cortical lesions	More cerebral involvement, often relapsing
**Brainstem Syndromes**	Occasionally present	Ataxia, diplopia, vertigo	May overlap with vascular comorbidities
**Atypical Presentations and comorbidities**	Rare	Intracranial hypertension, cranial neuropathies	Coexisting autoimmune or vascular disorders
**Disease Course**	Often monophasic, good recovery	30–50% relapsing	50% relapsing, higher disability accumulation

**Table 2:** 2023 Proposed Diagnostic Criteria for MOGAD [1].

Component	Criteria
A. Core Clinical Demyelinating Event	At least one of the following: Optic neuritis * Myelitis † Acute disseminated encephalomyelitis (ADEM) ‡ Cerebral monofocal or polyfocal deficits § Brainstem or cerebellar deficits ¶ Cerebral cortical encephalitis (often with seizures)
B. Positive MOG-IgG Test	- Serum testing using a cell-based assay ‡‡ - Clear positive **: No additional features required - Low positive †† or positive without reported titre: Requires AQP4-IgG seronegativity * and * ≥ 1 supporting clinical or MRI feature - Seronegative but CSF positive §§: Requires ≥1 supporting clinical or MRI feature
Supporting Clinical or MRI Features	- Optic neuritis: Bilateral simultaneous involvement, longitudinal optic nerve involvement (>50%), perineural optic sheath enhancement, optic disc oedema - Myelitis: Longitudinally extensive lesion, central cord lesion or H-sign, conus lesion - Brain/brainstem/cerebral syndrome: Multiple ill-defined T2 hyperintense lesions in supratentorial/infratentorial white matter, deep grey matter involvement, ill-defined T2 hyperintensity in pons/middle cerebellar peduncle/medulla, cortical lesion with or without meningeal enhancement
C. Exclusion of Better Diagnoses	Must exclude alternative diagnoses such as multiple sclerosis, AQP4+NMOSD, or other mimics ¶¶

* Optic neuritis: Unilateral or bilateral visual loss with pain, supported by MRI or exclusion of other causes. † Myelitis: Acute motor/sensory/sphincter dysfunction with MRI or CSF evidence. ‡ ADEM: Polyfocal deficits with encephalopathy and multifocal T2 lesions. § Cerebral deficits: Associated with T2 lesions in characteristic locations. ¶ Brainstem/cerebellar: Clinical deficits with corresponding MRI lesions. ** Clear positive: Live CBA ≥ 2 dilutions above cutoff or fixed CBA titre ≥ 1:100. †† Low positive: Live CBA in low range or fixed CBA titre 1:10–1:99. ‡‡ Serum testing is preferred; CSF testing only in select cases. §§ CSF positive: Valid only if serum is negative and clinical suspicion is high. ¶¶ Diagnosis requires clinical expertise to rule out mimics.

**Table 3:** Atypical Presentations of MOGAD.

Atypical Presentation	Key Features	Clinical Implications
**Overlap with Autoimmune Encephalitis** [25,26,27,28,29]	Co-occurrence with anti-NMDAR, less common with GFAP, CASPR2 antibodies; mixed demyelinating and encephalitic symptoms	Broad autoantibody testing in complex neuroinflammatory syndromes
**Atypical Ocular Inflammation** [31,34,35]	Scleritis, episcleritis, sclerouveitis, anterior uveitis (often steroid-responsive)	Consider MOG-IgG testing in recurrent or bilateral inflammatory eye diseases
**Intracranial Hypertension Syndromes** [36,37,38,39,40]	Headache, papilledema, visual field defects, may mimic or coexist with IIH; meningeal enhancement on MRI	Evaluate for MOGAD in atypical IIH cases, especially in non-obese patients or refractory cases
**Cranial Neuropathies** [41,42,43,44]	Facial palsy (CN VII), trigeminal neuralgia (CN V), vertigo/hearing loss (CN VIII), diplopia (CN III/IV/VI)	Test for MOG-IgG in inflammatory cranial neuropathies with brainstem involvement at root entry zone (REZ)
**Aseptic Meningitis/Hypertrophic Pachymeningitis** [22,45,47,48]	Headache, fever, neck stiffness, CSF pleocytosis; may mimic infectious meningitis	Consider MOGAD in recurrent or steroid-responsive aseptic meningitis, HaNDL syndrome (Headache with Neurologic Deficits and CSF Lymphocytosis) or Idiopathic Hypertrophic Pachymeningitis

**Table 4:** Acute treatment strategies in MOGAD.

Phase	Treatment	Notes
**Acute Attack** [49,50,51,52,53,54]	IV Methylprednisolone (1 g/day × 3–5 days)	Initiate within 24–72 h; monitor for steroid side effects
	Oral corticosteroid taper (2–3 months)	Prevents early relapse; longer taper than AQP4+NMOSD
	Plasma Exchange (PLEX)	For severe or steroid-refractory cases
	Intravenous Immunoglobulin (IVIG)	Alternative or adjunct to PLEX
**Refractory Cases** [49,50,51,52,53,54]	PLEX + IVIG combination	Consider in fulminant presentations
	Anti-IL-6 agents (e.g., tocilizumab)	Off-label; emerging evidence

**Table 5:** Off-Label Maintenance Therapies in MOGAD [49,50,51,52,53,54].

Therapy	Mechanism	Monitoring
Rituximab	B-cell depletion	CD19/20 counts, IgG levels
Mycophenolate mofetil	T/B-cell suppression	CBC, LFTs, pregnancy testing
Azathioprine	Purine synthesis inhibition	CBC, LFTs, TPMT testing
Monthly IVIG	Broad immunomodulation	Renal function, IgA deficiency
Tocilizumab/Satralizumab	IL-6 receptor blockade	CBC, LFTs, lipid profile, TB screening
**Avoid in MOGAD** [50,51]	Interferon-beta, Natalizumab, Fingolimod, MS DMTs	May worsen the disease or be ineffective

**Table 6:** Components of MOG-AR Score [76].

MOG-AR Risk Factor	Points
Onset age: 45 years or older	2
Sex: Female	2
*Attack phenotype at onset*	
Cerebral cortical encephalitis	4
ADEM	3
Optic neuritis	2
Cerebral monofocal or polyfocal deficits	1
Brainstem or cerebellar deficits	1
Myelitis	0
Immunosuppressive therapy: not used	5
Oral corticosteroids: <3 months	3
**MOG-AR score**	**%Risk (95% CI)**
0–4	33.3 (13.2–53.5)
5–8	43.1 (34.1–52.1)
9–12	70.5 (64.0–76.9)
13–16	78.8 (67.7–89.9)

**Table 7:** Relapse Risk Factors and Monitoring Tools in MOGAD.

Category	Factor/Tool	Clinical Implication
**Demographic** [1,3,8,21]	Age ≥ 45 years	Higher relapse risk (MOG-AR score)
	Female sex	Associated with increased relapse risk
**Clinical Presentation** [3,21,23,24,64,65,67,69,70,71]	Cortical encephalitis	Strong predictor of relapse
	ADEM or ON at onset	Moderate relapse risk
	Monophasic TM	Lower relapse risk
**Serological** [21,77,78,79,80,81,82,83]	Persistent MOG-IgG (>6 months)	Correlates with relapsing disease
	High MOG-AR score (≥9) [76]	Predictive of relapse (HR up to 3.29)
**Treatment History** [3,61,67,68,69,70,72,73,74]	No immunosuppressive therapy	Strongest predictor of relapse in the MOG-AR model
	Short IST duration (<12 months)	Associated with relapse after treatment discontinuation
**Biomarkers**	Elevated NfL, GFAP, MBP	Reflect active disease and axonal damage
**Imaging**	OCT: RNFL thinning [1,94,95,96,97,98,99,100,101]	Indicates optic nerve damage; useful for monitoring
	MRI: new or evolving lesions [1,86,87,88,89,90,91,92,93]	May detect subclinical activity; role in routine monitoring under investigation
**Monitoring Strategy**	Serial MOG-IgG titers [21,77,78,79,80,81,82,83]	May guide treatment duration and relapse risk stratification

## Data Availability

Not applicable.
